# Chatbots as frontline educators in sexual reproductive health rights: evidence, limitations, and ethical considerations

**DOI:** 10.3389/fdgth.2026.1777667

**Published:** 2026-03-19

**Authors:** Chioma Uzoma, Goldie Chukwuedo, Ejeh Isaac, Victory Uzoma, Stanley Chinedu Eneh, Felix Peace Chinyere, Oluchi Mmesoma Precious, Francisca Ogochukwu Onukansi

**Affiliations:** 1 Medvax Health, FCT, Abuja, Nigeria; 2 Community Health Department, Faculty of Clinical Sciences, Obafemi Awolowo University, Osun State, Ile-Ife, Nigeria; 3 Interdisciplinary Health Sciences, University of Texas at El Paso, El Paso, TX, United States; 4 Faculty of Nursing Science, University of Benin, Benin City, Nigeria; 5 Department of Pharmacy, Nnamdi Azikiwe University, Awka, Anambra State, Nigeria; 6 Department of Public Health, Federal University of Technology Owerri, Owerri, Imo State, Nigeria

**Keywords:** artificial intelligence in health care, chatbots, digital health, ethics, data privacy, health education, sexual and reproductive health and rights

## Abstract

Chatbots are increasingly used in digital health to expand access to information and support user engagement. In sexual and reproductive health and rights (SRHR), where stigma, privacy concerns, and health system constraints often limit timely access to accurate information, chatbots have been proposed as scalable tools for delivering education and facilitating service navigation. This perspective examines the role of chatbots as frontline educators in SRHR, drawing insights from existing evidence to assess the effectiveness of chatbots, practical implications, key limitations, and ethical considerations. Available evidence suggests that chatbots can enhance access to SRHR information, support user engagement, and contribute to improved knowledge, confidence, and linkage to services. However, the current evidence base remains uneven, with limited rigorous evaluation of long-term behavioural or health outcomes. Challenges related to accuracy, contextual responsiveness, privacy, equity, and accountability persist, underscoring the need for careful design and governance. Positioning chatbots as complementary components within integrated SRHR strategies, rather than standalone solutions, may offer a pragmatic pathway to harness their potential while safeguarding user rights and health outcomes.

## Introduction

Digital health technologies are reshaping how health education and information are delivered, with chatbots emerging as one of the most rapidly adopted tools for scalable, user-centric engagement (1). These conversational agents, ranging from simple rule-based systems to advanced artificial intelligence models, can provide on-demand, tailored responses to users’ queries without direct human mediation (2). Chatbots can be broadly grouped into rule-based and AI-enabled systems. Rule-based chatbots operate through predefined scripts and decision trees that trigger fixed responses to specific user inputs, making them suitable for structured education, frequently asked questions, and service signposting but less adaptable to complex or sensitive discussions; for example, Tess delivers psychoeducational content through scripted conversational pathways and has been evaluated as a rule-driven mental health support agent (3, 4). In contrast, AI-enabled chatbots use natural language processing and machine-learning models to interpret free-text input and generate context-responsive replies, allowing more flexible and personalized interaction, as demonstrated by conversational agents such as Woebot and large language model–based systems like ChatGPT. Recent evidence also shows that prompt-tuned AI chatbots can outperform base models in delivering sexual health information, while still remaining prone to occasional errors, highlighting the need for human oversight (5, 6). Some chatbots combine rule-based logic with AI-driven features, forming a hybrid category that leverages structured guidance while allowing limited adaptive interaction, particularly in SRHR contexts (7).

Within sexual and reproductive health and rights (SRHR), where stigma, privacy concerns, and provider shortages often limit access to timely information, the potential for chatbots to bridge gaps in knowledge and support is especially compelling (8). A Synthesis of digital healthcare literature suggests that chatbots can serve as promising interventions for SRHR information and service delivery because they offer anonymous, responsive conversation and linkage to services where appropriate, though they remain underdeveloped in many contexts and require careful integration with broader health systems (8–10). Empirical work in adjacent domains of health education supports the notion that AI-powered chatbots may improve engagement and promote health behaviors (11). Consequentially, a systematic review has found modest but positive effects of chatbots on health behavior change, including lifestyle and wellness outcomes, and highlight features such as personalization and 24/7 availability as key drivers of user engagement (12). In the mental health space, evidence indicates that chatbots can reduce distress and support behavior change among adolescents and young adults, though their effectiveness varies by design and context and rarely surpasses traditional intervention approaches (13).

Despite these promising signals, the evidence base remains fragmentary and context-dependent. Studies of SRHR chatbots reveal mixed perceptions among health professionals about their usefulness, with acceptance higher for general information and signposting but lower for complex counselling or emotional support (14). Moreover, questions about accuracy, equity, privacy, and integration into existing health services persist across digital health domains, underscoring the need for critical evaluation of both intended benefits and unintended consequences (15). This perspective therefore, examines the evidence on effectiveness, practical limitations, and ethical considerations of deploying chatbots as frontline educators in SRHR, with an emphasis on aligning technological innovation with health outcomes and community needs.

## Role of chatbots in sexual reproductive health right education

Chatbots in sexual and reproductive health and rights (SRHR) are digital conversational agents designed to simulate human dialogue and deliver tailored health information and guidance. These systems range from rule-based interfaces to advanced AI-driven chatbots capable of interpreting user input and offering context-specific responses (9). In digital health research, conversational agents are recognized for enhancing access to information and supporting patient engagement, particularly where traditional resources are limited or considered taboo (16).

One of the principal roles of chatbots in SRHR education is to offer anonymous and nonjudgmental access to sensitive information (17). Evidence from a realist synthesis, indicates that individuals are more likely to engage with SRHR content when they can interact with a chatbot without social or interpersonal judgment, especially in contexts where sexual health discussions are stigmatized. These interactions help users disclose personal concerns that might otherwise remain unspoken in face-to-face consultations, thereby facilitating initial empowerment and knowledge seeking (9).

Closely associated with anonymity is a chatbots’ ability to provide responsive and conversational information delivery. Unlike static content, chatbots can present complex SRHR topics, such as contraception options, sexually transmitted infection (STI) prevention, or consent frameworks, in an interactive sequence that adapts to user questions and follow-ups (9, 10). Evidence suggests that such conversational structures can increase user understanding and engagement, making health education more accessible and digestible (9, 18, 19).

Chatbots also play an important role in guiding users through SRHR service pathways. Several implementations integrate educational dialogue with signposting functions, such as directing users to nearby clinics, testing services, counselling resources, or appointment booking systems. Evidence from pilot deployments shows that chatbot interactions can prompt users to take concrete next steps toward care, positioning these tools as bridges between information access and service utilization (20).

Beyond individual information exchange, chatbots are increasingly embedded within broader digital health ecosystem**s**, including mobile health applications, youth-friendly platforms, and clinic-based digital interfaces. Their integration allows SRHR programs to extend reach beyond physical facilities and standard operating hours, while maintaining consistency in messaging and alignment with established health guidelines (1, 9).

## Evidence on chatbot effectiveness

Empirical research on the effectiveness of chatbots as educational or behavioral interventions in sexual and reproductive health and rights (SRHR) remains limited, but emerging evidence provides initial insights into their influence on knowledge, attitudes, and behaviors (10). For instance, a mixed-methods study of a pleasure-oriented SRHR chatbot deployed with young adults in Kenya found statistically significant improvements in users’ confidence discussing contraception (*P* ≤ 0.02), confidence discussing sexual feelings and needs (*P* ≤ 0.001), and ability to exercise sexual rights (*P* ≤ 0.01) after engaging with the chatbot. Participants valued the chatbot as a confidential and judgment-free source of information, and qualitative data indicated increased sex-positive communication and safer practices following interaction (21). Comparable digital interventions exist in both high-income and LMIC settings. For example, *Ask Roo* and *Layla's Got You* have engaged millions of conversations on contraception, STIs, and healthy relationships among adolescents and young adults in the United States, serving as widely cited models of chatbot-mediated SRHR education and signposting (10). Also, the SnehAI chatbot in India was designed to support adolescents and young adults with culturally tailored SRHR information and demonstrated strong engagement and accessibility features in program evaluation research (22). In addition to SnehAI, chatbots like *Tina* and a pleasure-positive bot documented in systematic reviews illustrate implementations in Uganda and other LMICs, offering contraceptive information and behavior support to broader audiences (10).

Additionally, the effectiveness of chatbot is also noted in other health domains, for instance, a systematic review and meta-analysis of chatbot-delivered interventions on vaccination attitudes found that tailored chatbot interactions led to significant improvements in attitude metrics compared to non-tailored approaches, although effects on actual vaccination intentions were mixed (23). Similarly, comprehensive reviews of AI chatbot interventions in women's health indicate that chatbot use was associated with improvements in psychological and behavioural outcomes, including reductions in anxiety and enhancements in engagement with health information, across multiple studies including randomized controlled trials and experimental designs. These findings suggest that chatbot interventions can influence intermediate outcomes relevant to SRHR education (24). More broadly, research on SRHR chatbot interventions across multiple countries highlighted that such systems are being implemented in diverse geographic and health-system contexts, including high-income and low-income settings, with varying levels of integration into service delivery and education programs (9).

It is worthy to note that, pilot implementation data also speak to the feasibility and uptake of chatbot application. In a pilot rollout of an AI chatbot for sexual and reproductive health information in clinical and community settings, the system recorded 1,749 user queries from 425 unique users over nine months, and around 10% of users went on to schedule a clinic appointment after interacting with the chatbot (20). While this study was not designed as a randomized efficacy trial, it demonstrates real-world engagement and suggests potential linkage between chatbot interaction and health service utilization. Similarly, recent evaluations using real-world clinical sexual-health questions show that chatbot performance can vary substantially depending on model design and training, reinforcing the need for careful evaluation of accuracy and safety when deploying SRHR chatbots in practice (6).

Reviews specifically focused on SRHR chatbots emphasize that, although evidence supporting definitive effects on health outcomes is limited, chatbots are promising interventions for delivering responsive information and supporting service access (9, 10). Overall, existing empirical work shows early signals of effectiveness in improving SRHR-related knowledge, attitudes, and service linkage, but also highlights that rigorous evaluation, particularly controlled trials with health behavior or clinical outcomes, remains sparse (23). This calls for more standardized evaluation frameworks, larger sample sizes, and longer follow-up to better assess how chatbot interventions compare to traditional health education strategies and how they influence sustained behavior change.

## Practical implications for using chatbot in SRHR programs

Emerging evidence, though limited, points toward several practical considerations for integrating chatbots into sexual and reproductive health and rights (SRHR) programs. First, chatbots can serve as scalable tools for expanding access to reliable SRHR information, particularly in settings where stigma or limited provider availability constrains traditional channels (9, 25). Evaluation studies using real clinical queries found that prompt-tuned conversational agents achieved high levels of correctness and safety in delivering sexual health information, with one AI chatbot outperforming a base language model in key measures including accuracy and provision of necessary information. This suggests utility for chatbots to supplement existing client education systems by offering immediate, guideline-aligned responses to common SRHR questions (6).

Programs aiming to increase knowledge and empowerment among target populations may also find value in chatbots designed to influence attitudes and self-efficacy. A study from Kenya reported statistically significant improvements among young adults in confidence discussing contraception, sexual rights, and sexual feelings after interacting with a pleasure-oriented SRHR chatbot. Participants described increased access to trustworthy, on-demand information and reported positive shifts in communication with partners, a practical outcome that community-focused education programs can leverage (21). Secondly, from a service integration perspective, chatbots can function as navigational bridges between information and care access. Real-world feasibility data from a clinic-based AI chatbot showed measurable linkage to services, with approximately one-tenth of users proceeding from chatbot interaction to schedule clinical care. This indicates potential for chatbots to support demand generation for SRHR services and to embed them as pre-visit engagement tools within service delivery pathways (20).

Thirdly, for programs prioritizing quality control and safety, broader systematic evidence from women's health research indicates that chatbot interventions can have measurable effects on health-related outcomes, such as reducing anxiety and improving engagement with care processes. Although this evidence is not SRHR-specific, it underscores the feasibility and potential utility of AI chatbots to support psychosocial and cognitive aspects of health education when coupled with appropriate oversight (24). Broader evidence of chatbot-based health interventions indicates that evidence for sustained behavioural change remains mixed, with effects varying across outcomes and intervention designs. This suggests that SRHR chatbots are likely to be most effective when integrated within multicomponent strategies, such as referral pathways or complementary digital and community-based interventions, rather than deployed as standalone solutions for complex behavioural change (26). Therefore, SRHR programs considering chatbot integration should prioritize domain-specific content adaptation, expert-informed quality assurance, and thoughtful integration within existing service delivery and referral systems.

## Key chatbot limitations

Despite their potential, chatbots used in health education, face notable limitations related to safety, communication capacity, and evaluation quality. A study revealed that chatbots highlight persistent gaps in handling complex or high-risk interactions and point to a broader lack of rigorous randomized trials and standardized evaluation frameworks, which constrains confidence in sustained impact and generalizability of findings (27, 28). Firstly, a key limitation concerns the accuracy and reliability of chatbot output, particularly in complex or ambiguous health contexts. One evidence-based study indicates that AI chatbots may generate imprecise or inappropriate information and are susceptible to producing plausible but incorrect responses, underscoring the need for domain-specific validation and human oversight to ensure safe use (24). Additionally, hallucinations, where generative conversational models produce confident but fabricated or misleading health information, particularly when responding to sensitive or context-dependent SRHR queries is another concern. Such risks are reported in recent evaluations of AI-driven health chatbots, highlighting the importance of safeguards such as curated medical knowledge integration, response verification layers, and escalation pathways to human professionals (6, 29).

Secondly, another important constraint is privacy, data security, and user trust. Empirical analyses of health chatbot applications reveal significant gaps in privacy protections, with many widely used apps lacking transparent privacy policies, limited options for users to control data sharing, and minimal safeguards against unauthorized access or misuse of sensitive health information. These issues have implications for user trust and ethical compliance, especially in SRHR where disclosures may involve highly sensitive personal details (30, 31). Concerns about data handling may also influence how users interact with chatbots, including willingness to disclose sensitive information or continue engagement, thereby affecting both safety and effectiveness (32). In addition, limitations in communication and contextual understanding may also hinder chatbot effectiveness.

Thirdly, a similar study (33), suggests that chatbots can struggle with complex language, cultural nuance, and context-specific cues essential to SRHR education, and that insufficient integration of behaviour-change theory and cultural adaptation may limit user engagement and relevance. Importantly, these limitations may differ depending on chatbot architecture. Rule-based systems or hybrid chatbots connected to verified health knowledge libraries may provide more consistent factual accuracy but reduced conversational flexibility. In contrast, fully generative AI chatbots may enable more natural interaction yet carry higher risks of misinformation, hallucinated responses, or context errors unless appropriately constrained. Recent work demonstrates how hybrid designs combining generative dialogue with structured health databases can improve reliability and safety of outputs (34, 35).

However, accessibility and equity concern further limit the reach of chatbot solutions. Uneven access to smartphones, reliable internet connectivity, and digital literacy across and within populations may exacerbate existing disparities in health information access (28). Finally, the ethical and accountability landscape for chatbot deployment remains underdeveloped. There are ongoing questions about responsibilities for harm when machine-generated advice leads to adverse outcomes, and few established regulatory frameworks clearly delineate accountability for errors or misinformation as argued by Kooli (36). These concerns highlight the need for explicit safety safeguards in chatbot deployment, including transparency about chatbot limitations, monitoring for harmful responses, audit trails, and clear referral mechanisms to qualified providers when risks are detected (36).

## Ethical and privacy considerations

Beyond technical limitations, the use of chatbots in SRHR raises important ethical and privacy concerns. Reviews of health chatbots indicate that privacy and data security are often insufficiently addressed, with many studies failings to report clear safeguards, data governance practices, or compliance with privacy regulations, highlighting a gap between technological deployment and responsible practice (37). Empirical evidence also highlights the role of user trust and privacy perceptions in chatbot adoption. Studies in digital health show that concerns about data privacy can reduce engagement and willingness to disclose sensitive information (38), a dynamic that is particularly salient in SRHR contexts involving intimate personal details.

Beyond privacy, the ethics literature on AI in health care further highlights accountability, transparency, and preservation of human-centred care as key concerns. Unclear responsibility for AI-generated guidance, limited transparency, and over-reliance on automated systems pose ethical risks, underscoring the need for clear disclosure and oversight (39, 40), particularly in sensitive SRHR context. Additional work in digital health ethics reviews underscores the need for procedural safeguards around informed consent and data governance. Users should be made aware of how their information is handled, what data is collected, and how it might be used. Transparent communication about data practices, coupled with robust encryption and adherence to relevant legal standards, are foundational to maintaining confidentiality and autonomy in sensitive health domains (41, 42). Additionally, User experience research indicates that trust in AI health chatbots extends beyond technical performance to include emotional and relational factors. While users may value accessibility, concerns about relational distance and privacy persist, highlighting the importance of ethical design that incorporates transparency, empathy, and respect for user autonomy (43).

## Future directions

Future research and practice on chatbots as frontline educators in sexual and reproductive health and rights (SRHR) should move beyond proof-of-concept deployments toward rigorous evaluation, ethical governance, and context-responsive design. Although early evidence suggests potential benefits for access, engagement, and education, multiple reviews highlight that the current evidence base remains fragmented and methodologically uneven, limiting conclusions about long-term effectiveness and scalability (26). First, there is a clear need for robust effectiveness studies using standardized outcome measures. Systematic reviews of health chatbot interventions consistently note a shortage of randomized controlled trials and longitudinal designs, with many studies relying on short-term self-reported outcomes or usability metrics (27). Future SRHR-focused research should prioritize experimental and quasi-experimental designs that assess not only knowledge gains but also sustained effects on attitudes, service uptake, and informed decision-making, while accounting for contextual and demographic variation.

Second, theoretical grounding and intervention transparency should be strengthened. Reviews of digital and conversational health interventions indicate that many chatbot systems lack explicit integration of established behaviour-change or educational theories, making it difficult to interpret mechanisms of action or replicate successful models (32). Embedding SRHR chatbots within clearly articulated conceptual frameworks, such as health literacy, empowerment, or social cognitive models, would enhance interpretability and guide more systematic development. Additionally, future directions should emphasize equity-centred and context-sensitive design, particularly for low- and middle-income settings. Evidence from global digital health research suggests that digital interventions may inadvertently reinforce existing inequalities when access, language, cultural norms, and digital literacy are insufficiently addressed (27, 44).

Notably, advancing the field will require clearer governance, accountability, and regulatory frameworks for AI-enabled SRHR tools. Ethics-focused analyses of AI in health care emphasize the absence of clear responsibility structures when automated systems deliver health information, particularly in sensitive domains (40). Future work should explore governance models that define accountability for content accuracy, data protection, and harm mitigation, alongside transparent disclosure of AI use and limitations to users. Finally, future research should investigate hybrid models that integrate chatbots with human-led SRHR services. Exploring models where chatbots support triage, education, and navigation while maintaining clear pathways to human support may offer a pragmatic balance between scalability and ethical responsibility.

## Conclusion

Chatbots show promise as complementary tools for delivering SRHR education and facilitating access to services, particularly in contexts shaped by stigma and limited provider availability. While early evidence indicates benefits for information access and user engagement, significant limitations and ethical considerations remain. Advancing their role in SRHR will require rigorous evaluation, equity-centred design, and robust governance to ensure that technological innovation aligns with rights-based and health system priorities.

## Data Availability

The original contributions presented in the study are included in the article/Supplementary Material, further inquiries can be directed to the corresponding author.
